# Adult spinal cord ependymal layer: a promising pool of quiescent stem cells to treat spinal cord injury

**DOI:** 10.3389/fphys.2013.00340

**Published:** 2013-11-28

**Authors:** Elena Panayiotou, Stavros Malas

**Affiliations:** Developmental and Functional Genetics Group, The Cyprus Institute of Neurology and GeneticsNicosia, Cyprus

**Keywords:** stem cells, spinal cord injuries, stem cell therapy, ependymal cells, central canal

## Abstract

Spinal cord injury (SCI) is a major health burden and currently there is no effective medical intervention. Research performed over the last decade revealed that cells surrounding the central canal of the adult spinal cord and forming the ependymal layer acquire stem cell properties either *in vitro* or in response to injury. Following SCI activated ependymal cells generate progeny cells which migrate to the injury site but fail to produce the appropriate type of cells in sufficient number to limit the damage, rendering this physiological response mainly ineffective. Research is now focusing on the manipulation of ependymal cells to produce cells of the oligodendrocyte lineage which are primarily lost in such a situation leading to secondary neuronal degeneration. Thus, there is a need for a more focused approach to understand the molecular properties of adult ependymal cells in greater detail and develop effective strategies for guiding their response during SCI.

Neural stem cells (NSCs) are specialized progenitor cells that can self-renew and generate both neurons and glia in the central nervous system (CNS). The first isolation of NSCs from adult brain lunched a new field of studies in neuroscience (Doetsch et al., 1997, 1999; Johansson et al., 1999). Adult somatic stem cells in general contribute to tissue homeostasis in all organisms as they support on-going tissue regeneration, replacing cells lost due to natural cell death or injury (Pellettieri and Sanchez Alvarado, 2007). Their function is required throughout life and their number needs to be maintained through a balance between self-renewal and differentiation. The underlying mechanisms that control this delicate balance are fundamental to understanding stem cell regulation and the therapeutic use of stem cells in human disease.

Adult NSCs have now been discovered in the two principal neurogenic regions, the hippocampus and the sub-ventricular zone (SVZ) of the forebrain (Doetsch et al., 1997; Bonaguidi et al., 2012). Their discovery raised the possibility that the CNS harbors regenerative power. The principal function of SVZ stem cells, however, is to replace lost neurons in the olfactory epithelium, while hippocampal stem cells are involved in memory.

The spinal cord in mammals is the least complex component of the CNS. In adult mice neurogenesis is not taking place. Only myelinating Oligodendrocytes (OLs) are produced from OL progenitors (OLPs) scattered throughout the parenchyma (Miller, 2002). Several studies in mice and humans confirmed that cells lining the central canal of the spinal cord, forming the ependymal layer, have stem cell properties *in vitro* and thus can generate multipotent neurospheres in culture (Weiss et al., 1996; Johansson et al., 1999; Dromard et al., 2008; Alfaro-Cervello et al., 2012; Bauchet et al., 2013). Under physiological conditions these cells are effectively quiescent and some proliferation that is taking place is restricted to ependymal cells located mainly on the dorsal tip of the central canal (numbering no more than 4–5 cells per 10 μM) (Meletis et al., 2008; Barnabe-Heider et al., 2010). During postnatal development, symmetric divisions of ependymal cells contribute to the elongation of the canal (Sabourin et al., 2009; Alfaro-Cervello et al., 2012). Likewise, adult ependymal cells divide symmetrically but all daughter cells remain in the ependyma, suggesting that their primary role in adults is ependymal cell maintenance (Barnabe-Heider et al., 2010).

While all NSCs in the SVZ of the forebrain express the stem cell markers Nestin, Sox2 and GFAP, in the spinal cord only dorsally situated ependymal cells express Nestin/GFAP, while Sox2 is expressed in all ependymal cells (Sabourin et al., 2009; Barnabe-Heider et al., 2010; Alfaro-Cervello et al., 2012). This implies that ependymal cells are most likely heterogeneous in their NSC potential and that dorsally positioned ependymal cells may possess different properties. Nevertheless, lineage tracing experiments have conclusively shown that all neurosphere-forming capacity in the adult spinal cord originates from ependymal cells with no evidence of sub-ependymal cell contribution but is unclear whether all spinal cord ependymal cells have equivalent stem cell potential (Barnabe-Heider et al., 2010).

The fact that embryonic ependymal cells proliferate and contribute to canal elongation, while adult ones are effectively quiescent, raises the possibility that the properties of ependymal cells in new-borns are likely to be different than those in adults. Indeed, in a limited search for the expression of some transcription factors in the spinal cord, we found that GATA3 is expressed in a dynamic manner in ependymal cells of the spinal cord as maturation proceeds and is not expressed in ependymal cells of other ventricles (data not shown). It is also intriguing that the Gata3 gene has never been found to be expressed in any CNS progenitors but only in some differentiated neurons. In the spinal cord, Gata3 is expressed in V2b interneurons and its expression is regulated by Notch signaling (Del Barrio et al., 2007). Notch signaling plays a critical role in the ontogeny of forebrain ependymal cells (Carlen et al., 2009) but less is known about its function on the ontogeny of spinal cord ependymal cells. Nevertheless, the fact that there are differences between these two ependymal cell populations makes the investigation of the molecular properties of the spinal cord ependymal cells more intriguing. Furthermore, the two populations respond differently in situations of injury. In stroke induced injury, ependymal cells enter the cell cycle and become depleted (Carlen et al., 2009) whereas, after spinal cord injury (SCI), ependymal cells proliferate and their progeny becomes recruited to the injured site leaving behind an intact ependymal layer (Meletis et al., 2008; Barnabe-Heider et al., 2010). Such unique molecular characteristic of the spinal ependymal cells could prove important in modulating their response in situations of injury.

## Ependymal cells become activated following spinal cord injury

SCI is a major cause of irreversible paralysis and currently there are no effective treatments. Several studies have shown that *in vitro* expanded neural progenitors derived from ependymal stem cells, when implanted into the injured site, can contribute to the formation of functional neuronal circuits in experimental models. Furthermore, the transplantation of spinal cord-derived neural progenitors promotes functional recovery (Hofstetter et al., 2005). Such recovery is much enhanced when myelinating progenitors are used [reviewed by Franklin and Ffrench-Constant (2008)]. However, exogenous administration of neural progenitors is not a process that has so far shown great promise not only due to the technical complications involved but also due to safety issues stemming from dangers in relation to the fate of implanted cells and the risk of carcinogenesis.

Despite the quiescent existence of ependymal cells under normal conditions, in models of SCI they increase significantly their proliferation rate and their progeny get recruited to the injured site. Indeed, following SCI, the neurosphere-forming capacity of ependymal cells is greatly enhanced (Meletis et al., 2008; Barnabe-Heider et al., 2010). Thus, the activation of endogenous stem/progenitor cells after injury or neural degeneration is another approach to reduce the effects of neuronal damage. This view is reinforced by the fact that ependymal cells are only a few millimeters away from any injured site and fast migration of their progeny may prove effective for self-repairing the injury.

In the uninjured spinal cord the vast majority of proliferating cells represent OLPs. Following SCI, ependymal cells and astrocytes become activated to a much higher degree than OLPs (Barnabe-Heider et al., 2010). Despite the multilineage potential of activated ependymal cells *in vitro*, the progeny generated *in vivo* following SCI is restricted to mainly glial cells the vast majority of which represent GFAP^−ve^ astrocytes, as shown by Sox9 expression. Newly formed ependymal-derived astrocytes migrate primarily to the center of the lesion site leading to the formation of the glial scar, which prevents axon regeneration. OLPs give rise only to OLs (Barnabe-Heider et al., 2010).

SCI results in rapid loss of OLs leaving many axons partly denuded leading to loss of impulse propagation. In partial SC lesions, which are more common, the loss of OLs leads to secondary neurological dysfunction and neuronal degeneration (Grossman et al., 2001; Lytle and Wrathall, 2007). The adult spinal cord microenvironment has strong progliogenic cues and prevents effective neuronal differentiation even when spinal cord stem cells engineered to favor neurogenesis are transplanted in the injured cord (Hofstetter et al., 2005). Infusion of growth factors, such as EGF and FGF2, into the central canal is able to increase the proliferation of ependymal cells and improve functional recovery after SCI (Kojima and Tator, 2002; Martens et al., 2002), suggesting that manipulation of endogenous ependymal cells may alter their differentiation potential. Nevertheless, the most attractive approach is to develop strategies for effective remyelination following SCI. The goal should, therefore, be to use current knowledge on OL development and specification to either limit the damage to OLs or modulate endogenous activation of ependymal cells toward generating more OL to replenish those lost.

Most approaches for therapies following SCI concentrate on limiting inflammation and secondary cell death, while at the same time promoting the restoration of functional circuits. In experimental SCI models, axonal regrowth can be enhanced through the administration of Brain-derived neurotrophic factor (BDNF), an agent that promotes axonal regrowth, possibly by recruiting myelinating OLs [reviewed by Weishaupt et al. (2012)]. More recently a study has shown that by interfering with the interaction between proNGF and p75 using a small, non-peptide, molecule named LM11A-3 prevented OL loss, increased the number of myelinating axons and improved functional recovery in an experimental model of SCI. Importantly this molecule can cross the blood-brain barrier without obvious toxicity complications (Tep et al., 2013).

In parallel to pharmacological manipulation of specific type of cells to limit functional impairment, the modulation of endogenously recruited ependymal-derived cells toward the OL lineage either using genetic engineering or by pharmacological means has not yet been reported. The fast-growing knowledge on glial cell specification during embryonic development should aid in this direction. For instance, the study of the regulation of the expression of the transcription factor OLIG2 in adult spinal cord could prove very useful in this regard. OLIG2 is not expressed in ependymal cells but only in OLPs scattered throughout the parenchyma (Meletis et al., 2008; Sabourin et al., 2009). OLIG2 is necessary and sufficient to drive OLP specification in the embryonic cord. Consequently, loss of OLIG2 leads to the conversion of prospective OLPs to Astrocyte progenitors suggesting that, at least in the embryonic spinal cord, OLIG2 regulates the fate of otherwise bi-potent glial progenitors (Lu et al., 2002; Zhou and Anderson, 2002; Liu et al., 2007). Since OLIG2 is not expressed in ependymal cells, the activation of this gene (or downstream target genes) in ependymal cells following SCI could provide insights into the ability of the latter to contribute to the formation of myelinating OLs in sufficient numbers to improve functional recovery. Indeed, some studies in the adult brain have shown that intraventricular administration of known pro-Oligogenic factors in the embryo (Lu et al., 2002) can enhance OL specification from stem cells demonstrating that pharmacological manipulation of ependymal cells is a viable option for ameliorating the effects of SCI. Furthermore, in the acute experimental autoimmune encephalomyelitis (EAE) animal model for Multiple Sclerosis, priming of NSC to become OLPs by overexpression of OLIG2 resulted in improvement of clinical symptoms following intra-ventricular delivery of the engineered NSCs (Sher et al., 2012).

In summary, several studies on the properties and behavior of ependymal cells in models of SCI have converged on to a number of findings that could pave the way for more effective therapies. Of major importance is the recruitment of a large number of ependymal-derived cells at the injury site while at the same time maintaining the ontogeny of the canal. Equally important is the fact that the astrocytes produced are positioned in the center of the glial scar opening the way for a more targeted approach to manipulate the fate of activated ependymal cells. Crucial in these efforts is to develop strategies for initiating remyelination at the injury site by manipulating the fate of cells produced from activated central canal stem cells. Forcing these cells to initiate a pro-oligogenic pathway of glial specification should be at the center of future experimentation. Such effort should be aided through combined approaches using knowledge from embryonic development and cellular responses following tissue damage in SCI conditions. It is likely that in the next few years we will be confronted with exciting developments that will open the way for clinical trials to treat SCI.

### Conflict of interest statement

The authors declare that the research was conducted in the absence of any commercial or financial relationships that could be construed as a potential conflict of interest.
